# Bilateral symmetry of vertical time to stabilization in postural sway after double-leg landing in elite athletes with unilateral chronic ankle sprain

**DOI:** 10.1186/s13047-022-00552-5

**Published:** 2022-05-31

**Authors:** Ali Yalfani, Zahra Raeisi

**Affiliations:** 1 Department of Sport Rehabilitation, Faculty of Sport Sciences, Bu-Ali Sina University, Hamedan, Iran; 2 Department of Sport Rehabilitation, Faculty of Sport Sciences, Arak University, Arak, Iran

**Keywords:** Stability, Postural control, Soccer, Basketball, Injury prevention

## Abstract

**Background:**

Lower limb asymmetry among athlete with unilateral chronic ankle instability (CAI) during bilateral landing can be a potential source of ankle sprain reinjury. The aim of study was to investigate the effect of bilateral symmetry of vertical time to stabilization (vTTS) in postural sway after double-leg landing (DLL) in elite athletes with unilateral CAI.

**Methods:**

Twenty professional players with unilateral CAI and ten healthy controls were assigned to three groups (soccer, basketball, and control groups, *n* = 10 each). The postural balance during DLL tasks was assessed based on center of pressure (CoP) and vTTS. Multiple analysis of variance was conducted to statistically analyse the CoP and vTTS which followed by Bonferroni’s post hoc test (*P* < 0.05).

**Results:**

The vTTS of the injured foot was significantly longer in the soccer and basketball players than in the control players (*P* = 0.006, *p* < 0.001 respectively). The intragroup comparison showed a significant difference in the vTTS of CAI and uninjured feet among the basketball players (mean difference = 1.3 s). The basketball group exhibited a worse balance in CoP oscillations results between groups.

**Conclusions:**

The findings suggested that symmetry between double-leg vTTS values, may be important as much as the sooner vTTS in reduced CoP oscillations and enhanced balance after DLL. Balancing exercises should achieve sooner vTTS in soccer players and symmetry in the double-leg vTTS of basketball players with unilateral CAI while maintaining static balance during dynamic-to-static postural changes to reduce recurrent ankle sprain.

## Background

Ankle sprain is very common among basketball, and soccer players because of the special nature of these sports, which involve movements such as rapid changes in direction, frequent jumping and landing, sudden stops, and collisions with opposing players during rebounding, defending, and tackling [1, 2]. This type of injury accounts for 41.1% [3] and 14% [4] of the total number of injuries suffered by basketball and soccer players, respectively. Its initial occurrence can increase the odds ratio of repeat incidence by five times and is considered to be the strongest predictor of subsequent injury [2, 5]. Recurrent ankle sprain leads to chronic ankle instability (CAI), which it can causes an altered activation pattern of muscles, reoccurring harm, athletic and participatory limitations, and imbalance of posture [6, 7].

Landing has been recognized as one of the most common causes of non-contact ankle sprain and postural controlling after landing is one of the most important factors in preventing injury [8]. Some studies reported that the risk of ankle sprain in people with balance deficits is up to seven times higher than that in their healthy counterparts [9]. One of the important parameters of postural controlling during landing is the time to stabilization (TTS) [8]. TTS is a variable for measuring neuromuscular control [10], and vertical TTS (vTTS) refers to the time at which the vertical force of the ground after a dynamic disturbance is stabilized until the body reaches a steady posture. The ability to quickly stabilize is regarded as a positive and protective function of the body, but the interval at which this stabilization is achieved is longer in people with functional ankle instability [11]. Recurrent injuries and CAI impair somatosensory receptors, thereby affecting postural control and diminishing the ability of the body to carry out an appropriate motor response to maintain postural balance [12]. However, some researchers suggested that center of pressure (CoP) parameters are more sensitive in detecting postural control disorders in people at risk of developing ankle sprain [13]. In this regard McKeon and Hertel mentioned, the CoP excursion measured the 3-dimensional interaction between the foot and the force plate as the body attempt to maintain itself over a fixed base of support [13].

The effect of ankle sprain on balance control has been investigated in previous studies [14, 15]. Friden et al. observed a decrease in postural control in the injured and healthy limbs of athletes with acute ankle sprain compared with the observed levels in a reference group [14]. Furthermore, Evans et al. examined the role of acute lateral ankle sprain in balance and suggested the occurrence of defects in postural control in both injured and healthy limbs. Moreover, there have been indicated that defects in postural control were eliminated 7 days after injury to healthy organs compared to injured organs which remained at least 4 weeks after injury [15]. Despite the high scientific values of mentioned studies, however, insufficient information has been derived on how unilateral CAI affects defects in postural control compared with levels observed pre-injury in both injured and healthy limbs. There is also lack of data on the longevity of postural control under the aforementioned conditions. Consequently, defects in postural control given CAI appear to be more pronounced than defects that occur owing to acute sprain [13].

Most landing-related studies have assumed that the right and left lower limb are symmetrical [16, 17]. Therefore, these studies have only chosen the dominant side to represent the overall performance of the subjects’ bilateral lower extremities [8, 18]. However, considerable studies show that the presence of symmetry affects the lower extremities during landing movements [17, 18]. Previous studies showed significant differences in knee moment and joint range of motion between dominant and non-dominant leg during double-leg landing [16, 19]. The researchers declared that asymmetry in lower extremities should be a critical factor in studies because it can apply overload in one leg and this may undertake more unilateral injury in lower extremities [17]. Therefore, lower limb symmetry in double-leg landing tasks is a cornerstone assessment in studies. Hence, it is important to pay attention to bilateral symmetry in professional athletes with unilateral injuries.

Although previous studies compared differences in postural control among professional athletes with no history of injury or between injured athletes and healthy controls, to our knowledge, no study has differentiated balance in various professional athletes with unilateral CAI. Furthermore, most of previous researchers have studied vTTS during single-leg landing task. While most sports skills occur in double-leg position and single-leg landing task does not seem to give us enough information for achieve better balance after double-leg landing in athletes with unilateral CAI. In this respect, the purpose of this study was to compare the postural control abilities (CoP oscillations and vTTS) of professional soccer and basketball players with unilateral CAI and healthy control during the performance of a double-leg landing task. The current work hypothesized that postural control during a double-leg landing task differs among professional athletes with unilateral CAI. We also hypothesized that oscillations would be lower in the group with faster vTTS. The results could add additional information to the literature on the role of bilateral symmetry in the ability to maintain postural balance in elite athletes with unilateral CAI. Also, the results can adjustments in balance rehabilitation programs in accordance with the specific requirements of each sport and athlete.

## Methods

### Participants

A total of 30 professional male athletes were assigned to three groups of soccer (*n* = 10, ages [mean ± SD] = 23.9 ± 2.33) and basketball (*n* = 10, ages [mean ± SD] = 24.1 ± 2.3) with unilateral CAI and healthy control (*n* = 10, ages [mean ± SD] = 24 ± 2.66). The sample size was calculated using G*Power at an effect size of 0.5, a test power of 0.8, and an α value of 0.05. This study was carried out according to the Declaration of Helsinki and approved by the local Ethics Committee of Bu Ali Sina University (code IR.BASU.REC.1399.007). Before experimental procedures began, all the participants reviewed and voluntarily signed an informed written consent form. This study was performed in the Sports Rehabilitation Laboratory of Bu Ali Sina University (Hamedan-Iran) from September 10, 2020 to December 20, 2020.

In our study, professional athletes working in Premier League soccer and basketball teams were selected on the basis of the inclusion and exclusion criteria recommended by the International Ankle Consortium [20]. Specifically, the inclusion criteria for soccer and basketball groups were a history of ankle sprains requiring two rounds of medical treatment or more, a feeling of fear over instability in ankle function, collapse while performing physical activities, gaining a Cumberland Ankle Instability Tool (CAIT) score ≤ 24, and a confirmed severity in the functional instability of the ankle, as determined via anterior drawer and talar tilt tests performed by an experienced physician [6]. Finally, the athletes recruited for participation should have had CAI on right foot only, with the affected leg as the dominant one. The dominant leg was identified by the athletes or determined through a test that compelled the dominant leg to hit a soccer ball [6]. The exclusion criteria were a history of lower limb surgery and a history of fractures and acute damage to the musculoskeletal structure or joints of the lower limbs (e.g., sprains and fractures) in the past 3 months leading to at least 1 day of physical activity loss [20].

**Fig. 1:**
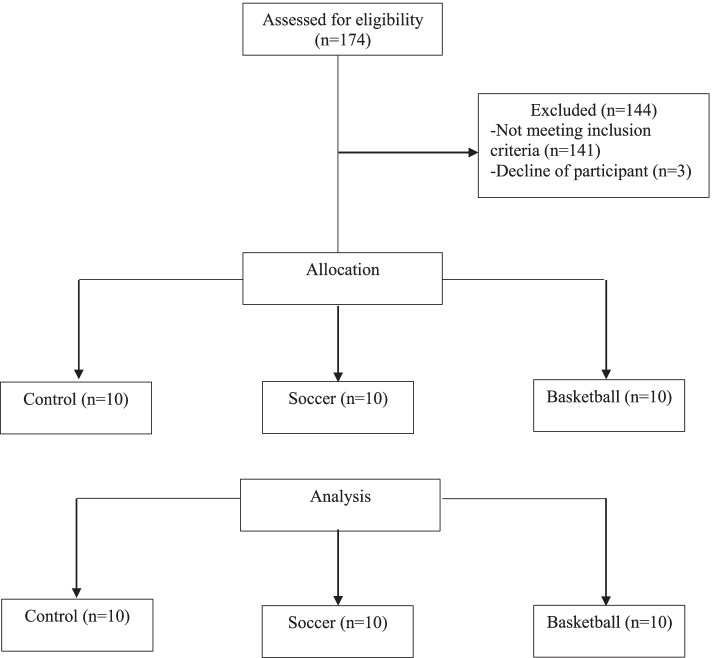
Flowchart of the study design

The participants in control group (5 professional soccer athletes and 5 basketball players) were healthy with no history of ankle or knee injury in the past 12 months, as well as no history of surgery or fractures of the lower extremities; chronic diseases, such as patellofemoral pain syndrome [21]. The right leg was dominant in control group athlete (Fig. 1).

### Experimental procedure

**Fig. 2:**
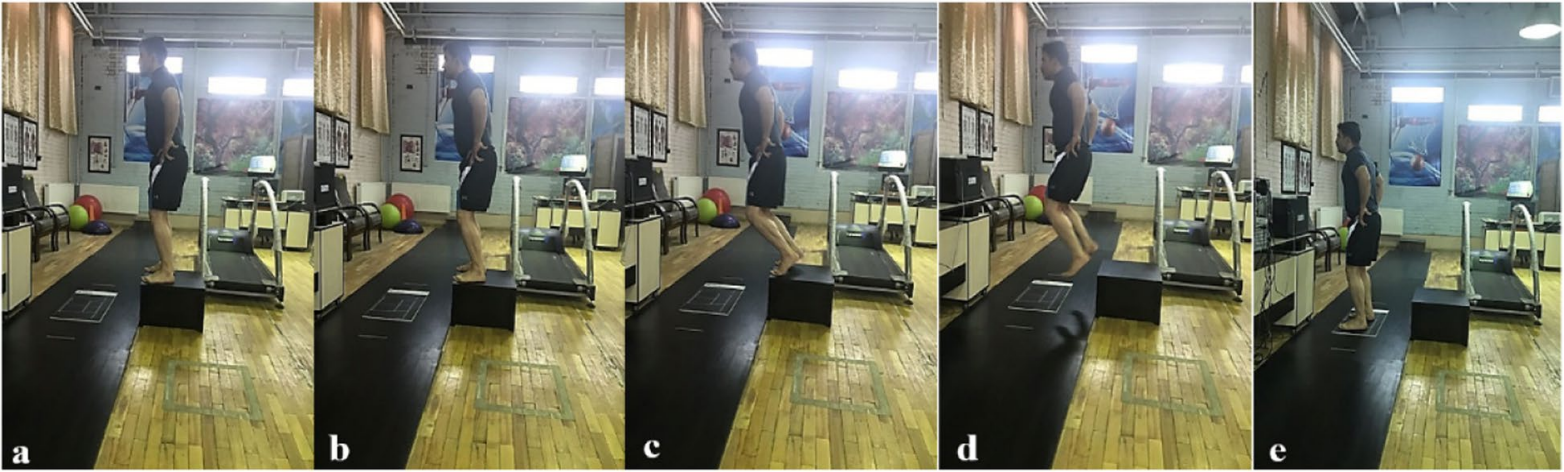
Phases of double-leg landing task from the step. **a** Standing on the step, **b** Preparing for landing, **c** Landing begins, **d** Flying in the air, **e** Landing

The tests were performed at the Sports Rehabilitation Performance Laboratory. At the first session, the participants signed an informed written consent form and completed questionnaires related to their histories of injury and CAIT scores. Initially a 15 minutes warm-up exercises including 5 minute running on the treadmill at the self-selected speed and stretching exercise focusing on the lower extremity were performed [6, 16], after which the participants were asked to perform a double-leg landing movement on a platform from a step that was 30 cm high and positioned 15 cm from the center of a plantar pressure platform. They were instructed landing without jump while regaining postural stability in the shortest possible time and maintaining the posture with the least possible oscillations for 10 seconds. No instructions were given to the participants on how to land; they were directed only to place their hands on their hips. All landings were completed in a double-legged manner and barefoot (Fig. 2). To familiarize themselves with the correct movement, the participants were given the opportunity to practice the target task three times before the tests were commenced. Finally, three successful trials with a rest time of 30 seconds were recorded between each landing for each participant [11, 12].

### Instrumentation

A plantar pressure platform (FDM-S, Zebris Medical GmbH, Germany) composed of 2560 high-sensitivity sensors and having an acquisition frequency of 120 Hz was used to record the vTTS and CoP trajectories during the double-leg, landing task.

### Data processing

The CoP oscillation parameters included the following items, which were measured 3 and 5 seconds after the completion of the target task:

CoP sway velocity (CoP SV) (displacement velocity of the CoP during trial).

**Fig. 3:**
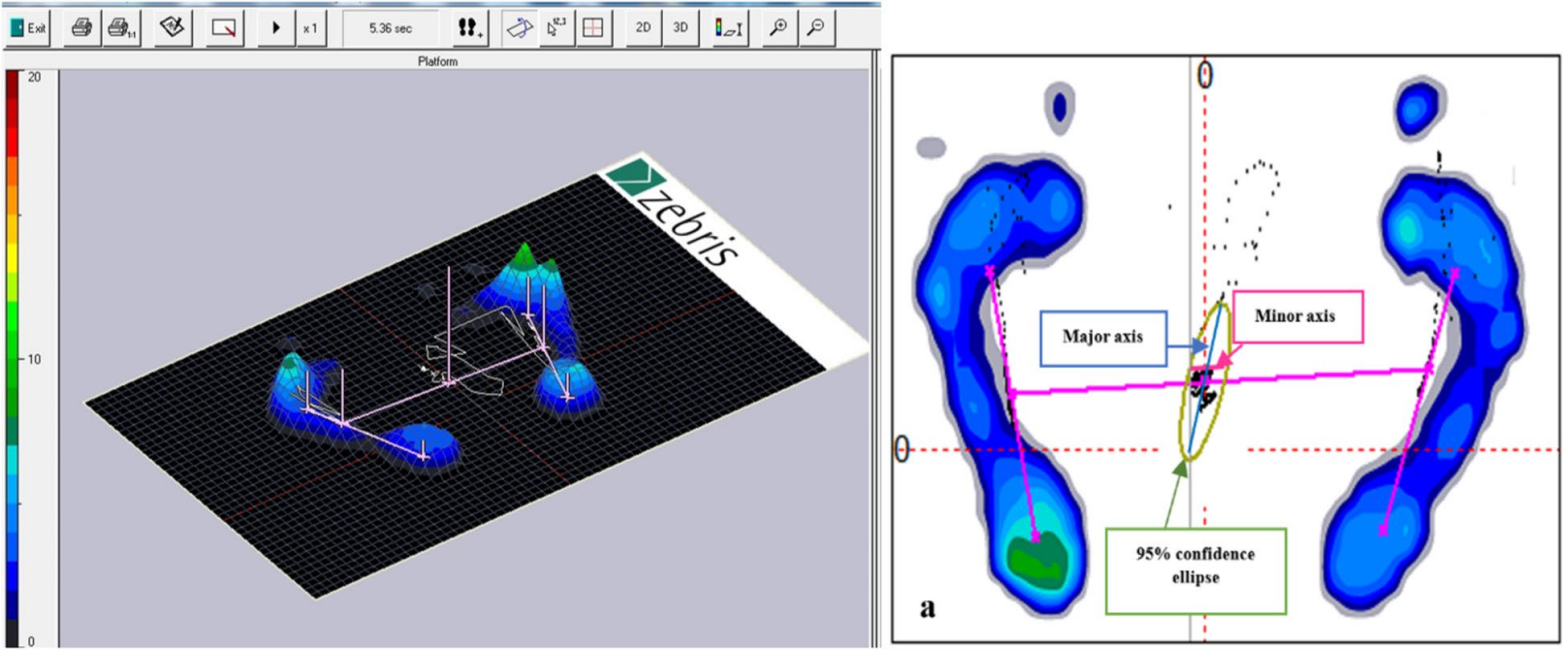
Win FDM-S software, the image shows the average load distribution under the feet. The color scale on the left quantifies the load distribution. **a** The horizontal pink line is the connecting line of the three CoPs (Center of pressure). The middle cross is the center of pressure of the whole body. The ellipse around includes 95% of the CoPs. Minor axis (CoP displacement in mediolateral direction), Major axis (CoP displacement in anteroposterior direction)

CoP displacement in the mediolateral (ML) (Minor axis) and anteroposterior (AP) (Major axis) directions. (Fig. 3).

The vTTS data was obtained in each test using WinFDM-S software (09.02.01 version). The trial data were then exported from the software, and the vTTS in each test was obtained using MATLAB (version R2010b, MathWorks, Natick, MA) following the procedure of Wright et al. (2016) with a slight difference [11]. Briefly, the data imported into MATLAB software were initially rectified and filtered with a 12 Hz second-order low-pass Butterworth filter. A normalized reference variable was calculated on the basis of the participants’ trial results; that is, the mean vGRF in the last 2 sec of each trial (8–10 seconds) was ascertained. Subsequently, three standard deviations from the mean for a range of normal variations were calculated for each participant in all the trials. An unbounded third ordinal polynomial with a rectified force of 10 seconds after landing was fitted for each participant and each trial. The vTTS was defined as the point at which the unbounded third ordinal polynomial exceeded the range of variation occurring in the first trial.

### Statistical analysis

The normality of the data was obtained using the Shapiro-Wilk test. Multiple analysis of variance (MANOVA) was conducted to statistically analyse the CoP and vTTS which followed by Bonferroni’s post hoc test. The relationship between vTTS and CoP parameters was determined by using Pearson correlation coefficient. Interpreting of correlations’ results executed based on Evans’ classification (1996) which indicated the values of r respectively between 0.00–0.19 (very weak), 0.20–0.39 (weak), 0.40–0.59 (moderate), 0.60–0.79 (strong), and 0.80–1.00 as a very strong correlation [22].

An alpha of 0.05 was used for all statistical test. All the analyses were performed using the Statistical Package for the Social Sciences (Version 21; SPSS Inc., Chicago, IL). In the statistical analysis of vTTS, the right foot of the soccer and basketball groups (injured-dominant foot) was compared with the right foot of the control group (dominant foot). Also, the left foot was compared in three groups.

## Results

The characteristics of the athletes are listed in Table 1.

**Table 1:** Athletes’ characteristics (mean ± standard deviation)

Groups	Control	Basketball	Soccer
Age (year)	24 ± 2.66	24.1 ± 2.3	23.9 ± 2.33
Height (cm)	187 ± 3.39	188.2 ± 3.08	187.5 ± 2.91
Body mass (kg)	77.2 ± 3.96	80.8 ± 5.07	80.7 ± 3.46
CAIT (score)	29.3 ± 0.82	20 ± 1.49	21.1 ± 1.1
Ankle sprains (number of times)	0	4.2 ± 0.78	4.1 ± 0.73

*CAIT* Cumberland Ankle Instability Tool

### vTTS and symmetry

The statistical analysis indicated a significant difference in the vTTS of the right (injured in soccer and basketball) foot was found among the groups (F2,54 = 10.39, *P* < 0.001, ŋ2 = 0.278). The Bonferroni’s post hoc test showed a significant difference in the vTTS of the right foot between the control group and the soccer (*P* = 0.006) and basketball (*P* < 0.001) groups. Also, in left foot a significant difference founded between the control and soccer groups (*p* = 0.023). (Table 2).

**Table 2:** Mean (± standard deviation) vTTS and COP variables of the soccer, basketball and control players and MANOVA results

		Soccer		Basketball		Control		F_**2,54**_	***p***	ŋ^**2**^	Post hoc
		M	SD	M	SD	M	SD				
vTTS (sec)	Right foot	1.64	1.13	2.05	1.32	0.49	0.19	10.39	< 0.001^*^	0.278	C < S C < B
	Left foot	1.49	0.76	0.74	0.36	0.5	0.15				C < S
	p	0.67		0.001^*^		0.973					
AP (mm)	3 Sec	29.78	3.87	34.38	6.69	21.66	3.09	28.34	< 0.001^*^	0.512	C < S C < B
	5 Sec	25.02	3.52	29.05	6.55	19.44	3.05				C < S C < B
	*p*	0.029^*^		0.015^*^		0.299					
ML (mm)	3 Sec	62.28	9.11	78.06	8.19	61.09	15.41	13.95	< 0.001^*^	0.341	C < B S < B
	5 Sec	51.89	8.33	63.91	2.78	51.9	10.63				C < B S < B
	*p*	0.022^*^		0.002^*^		0.041^*^					
SV (mm/sec)	3 Sec	121.61	14.64	146.7	22.59	119.87	18.7	10.19	< 0.001	0.274	C < B S < B
	5 Sec	79.23	10.68	92.86	14.07	78.84	12.98				–
	*p*	< 0.001^*^		< 0.001^*^		< 0.001^*^					

*vTTS* Vertical time to stabilization, *AP* Anteroposterior CoP oscillations (major axis), *ML* Mediolateral CoP oscillations (minor axis), *SV* Sway velocity of CoP, *C* Control group, *S* Soccer group, *B* Basketball group

* *p* < 0.05

The comparison of the uninjured and CAI feet of the groups showed a significant difference between the vTTS of the uninjured and CAI feet (F1,54 = 5.46, *p* = 0.023, ŋ2 = 0.092). The post hoc revealed that vTTS of the uninjured foot of the basketball players was significantly less than that of the CAI foot (*p* < 0.001, mean difference = 1.3 s). In the soccer (*p* = 0.679, mean difference = 0.14 s) and control (*p* = 0.973, mean difference = − 0.01 s) groups, no significant difference in vTTS values was found between uninjured and CAI feet.

### CoP displacement and velocity

A significant effect of main factors (group) (F6,106 = 12.20, *p* < 0.001, ŋ2 = 0.409) and (time) (F3,52 = 52.96, *p* < 0.001, ŋ2 = 0.75) on the CoP’s parameters was found. The groups revealed significant difference in AP, ML and CoP SV at 3 and 5 second after double leg landing (Table 2).

The MANOVA results on the CoP variables showed significant differences in the Major axis (AP) (F2,54 = 28.34, *p* < 0.001, ŋ2 = 0.512), Minor axis (ML) (F2,54 = 13.95, *p* < 0.001, ŋ2 = 0.341), and CoP SV (F2,54 = 13.95, *p* < 0.001, ŋ2 = 0.274) among the groups (Table 2). The Bonferroni’s post hoc test results showed a difference in the AP of CoP oscillations between the control group and the soccer (*p* = 0.001, *p* = 0.032) and basketball (*p* < 0.001, *p* < 0.001) groups respectively at 3 and 5 seconds after landing. The test also reflected a significant difference in ML of CoP sway between the control group and the soccer (*p* = 0.002, *p* = 0.025) and basketball (*p* = 0.001, *p* = 0.025) groups at 3 and 5 second after landing respectively. The post hoc analysis revealed a significantly faster CoP SV between the control group and soccer (*p* = 0.003) and basketball (*p* = 0.001) groups at 3 seconds after landing. But no significant difference was found between the CoP SV in study groups after 5 second of landing (*p* > 0.05).

The results of comparison of AP CoP oscillation in groups showed that the amount of sway has decreased over time at the third second to the fifth (F1,54 = 11.28, *p* = 0.001, ŋ2 = 0.173). Then, the results of the post hoc test showed a significant difference between the soccer (*p* = 0.029) and basketball (*p* = 0.015) groups. The control group did not show a significant difference (*p* = 0.299). Also, in all three groups, the ML CoP oscillations (F1,54 = 19.68, *p* < 0.001, ŋ2 = 0.267) and CoP SV (F1,54 = 121.05, *p* < 0.001, ŋ2 = 0.692) reduced significantly with the passage of time (time 5 seconds compared to 3 seconds). The results of the post hoc test are given in the Table 2.

### Correlation between vTTS and CoP parameters

Analyzing the data showed a strong correlation between vTTS of right and left foot in soccer and control groups. In the soccer group the significant correlation was found between the vTTS value of affected foot and AP of CoP oscillation at 3 second after landing (Table 3). Furthermore, except the ML CoP sway after 5 second, there were a significant and strong correlation coefficient between the vTTS value of affected foot and CoP parameters after landing in the basketball group (Table 3).

**Table 3:** Pearson correlation coefficient results

			AP 3 s	AP 5 s	ML 3 s	ML 5 s	SV 3 s	SV 5 s	vTTS L
Soccer	vTTS R	r	0.647	0.57	0.415	0.136	0.231	0.361	0.885
		*p*	0.043^*^	0.085	0.233	0.708	0.521	0.305	0.002^*^
Basketball	vTTS R	r	0.801	0.801	0.649	0.608	0.796	0.779	0.378
		*p*	0.005^*^	0.005^*^	0.042^*^	0.062	0.006^*^	0.008^*^	0.282
Control	vTTS R	r	0.168	0.144	0.239	0.316	0.322	0.302	0.796
		*p*	0.642	0.691	0.506	0.373	0.364	0.397	0.007^*^

*vTTS R* Vertical time to stabilization of right foot, *vTTS L* Vertical time to stabilization of left foot, *AP 3 s* Anteroposterior CoP oscillations (major axis) until to third second, *AP 5 s* Anteroposterior CoP oscillations (major axis) until to fifth second, *ML 3 s* Mediolateral CoP oscillations (minor axis) until to third second, *ML 5 s* Anteroposterior CoP oscillations (minor axis) until to fifth second, *SV 3 s* Sway velocity of CoP until to third second, *SV 5 s* Sway velocity of CoP until to fifth second, *C* Control group, *S* Soccer group, *B* Basketball group

* *p* < 0.05

## Discussion

Based on the aim of the present study which was to compare the bilateral symmetry of vertical time to stabilization and postural sway after double-leg landing in elite athletes with unilateral CAI. We hypothesized that postural control during a double-leg landing task differs among professional athletes with unilateral CAI. This supposition was confirmed by the vTTS results. The vTTS value of the right foot significantly differed among the groups; the control players exhibited faster vTTS than that displayed by the two other groups, and the soccer players had a faster vTTS than did the basketball players.

The functioning of the postural control system as a circuit for managing feedback between the brain and the musculoskeletal system reflects balance [9]. Proper neuromuscular control, which can be defined as the unconscious activation of dynamic limitations in preparation for and in response to the movement and loading of the joint to maintain and restore joint functional stability, is also one of the factors affecting postural control [10].

An intergroup examination of the dissimilarities in vTTS between the CAI and uninjured feet indicated a significant difference in the vTTS in the basketball group; a difference in mean vTTS values of the CAI and uninjured feet was (1.3 s) among the basketball group. In soccer (0.14 s), and control (− 0.01 s) groups this difference was not significant between feet. We also hypothesized that oscillations would be lower in the groups with faster vTTS. This hypothesis was confirmed by the results of the control group. In contrast to the vTTS of our results in soccer groups.

A faster TTS is considered a positive feature, whereas a greater CoP trajectory is usually interpreted as indicative of diminished stability [23]. Some of our notable findings are the significant difference in the vTTS of the uninjured foot and CAI foot in the basketball group and the minimum difference between the mean double-leg vTTS values in the control and soccer groups. The lower CoP oscillations in the soccer group can be attributed to the slight difference in vTTS values between the uninjured and CAI feet. The basketball players may have attempted to better control their balance in the post-landing period by increasing CoP oscillations, the difference between the two feet, and the difference in achieving bipedal stability. Optimal motor-postural tendencies that lead to potential imbalances, such as asymmetry between the lower limbs and postural oscillation defects, may expose athletes to injury and reduce performance [24].

According to our results, the soccer players performed better in controlling balance via CoP oscillations than did the basketball players. Researchers suggested that the repeat practice of professional athletes may alter balance owing to neurological adaptations [25]. One of the other reasons for this discrepancy may be the difference in techniques adopted by basketball and soccer players. Basketball players use both the upper and lower limbs to perform movements. They also frequently perform shooting, dribbling, and passing skills using the upper limbs. On the other hand, soccer players usually perform passing, shooting, and dribbling using the lower limbs with cleated or non-cleated shoes on the field [25]. They can improve balance by further enhancing proprioception through challenges to motor systems, in addition to fully engaging the lower limbs and their joints.

According to the finding of present study, a strong correlation demonstrated between the vTTS of injured and uninjured foot and also there were a correlation of the vTTS and AP in soccer group. On the other wise, the longer vTTS and higher AP sway are related to each other. This may be due to the weakness of the ankle strategy in the injured foot, which is responsible for controlling AP oscillations [26, 27]. In basketball group, except the ML at 5 second after landing, the injured vTTS was correlated with all of the CoP parameters.

It is unknown whether this difference is due to the initial dissimilarities of soccer from basketball or whether it is the result of unilateral CAI and that’s related to compensation mechanisms. The athletes with unilateral CAI, when asked to land in both feet, may protect the affected foot from symptoms of ankle instability. Perhaps the differences found in the injured and uninjured foot among the soccer group are related to compensation mechanisms. The studies showing delays in vTTS tested individuals with ankle instability during single-leg drop jumps [11]. These issues highlighted the important points such as the need for different plans for rehabilitation phases and designs of specialized exercises to improve of balance in professional athletes with unilateral CAI. For instance in soccer players, exercises should be designed in a way that helps athletes achieve vTTS in the shortest possible time to reduce the odds ratio of recurrent injury. It is best to use training programs that reduce the vTTS, such as those proposed by Ross and Guskiewics [28]. They suggested the method of coordination training (such as balance on foam, circular motion on a wobble board and resistance band kicks) with stochastic resonance stimulation as an adjunct therapy [28]. Exercise programs for basketball players should be including strengthen ankle and hip-oriented strategies for balance control in static positions, following a shift from dynamic to static tasks. Moreover, functional activities and range of motion training combined verbal feedback are recommended for lower extremities balance controlling following a shift from dynamic to static task [29, 30].

According to Lobo et al. bilateral impairment of postural control strategies in the participants with LAS must be considered [31]. Investigation body of literature regarding athletes’ landing with unilateral CAI, showed researchers often studied single leg landing and assumed the movement symmetry between a participant’s injured and uninjured limbs for the simplicity of data collection and analysis, while landing tasks often involve dual-limb motion [17]. However, it is suggested that future studies consider bilateral limb during the different dynamic task in elite athletes with unilateral CAI and don’t assume lower limb symmetry.

In this study, TTS was measured only in the vertical direction, but a more accurate approach would be to measure this variable in the AP and ML directions. The research involved only young professional male athletes with CAI, but different findings may be derived when other groups are recruited.

## Conclusions

The findings of our study make several outstanding contributions to the current literature. Firstly, the vTTS symmetry between foot during double-leg landing in elite athletes with unilateral CAI can be important as much as the sooner vTTS in reduce of postural sway after landing. In addition, increasing differences and longer vTTS in the injured foot in the basketball group were associated with SV, AP, and ML oscillations which can be a factor in the recurrence of ankle sprain injury in these people under competition pressure.

Training exercises appear to differ among various athletes with unilateral CAI. Thus, while planning balance rehabilitation exercises for basketball players, coaches and physicians should focus on exercises that develop symmetry in vTTS in both feet and maintain static balance, following a change in posture from dynamic to static conditions. For soccer players, balance exercises should be designed in a way that helps athletes achieve vTTS in the shortest possible time to reduce the odds ratio of recurrent injury.

## Data Availability

The datasets used and/or analyzed during the current study are available from the corresponding author on reasonable request.

## Abbreviations

CAI: Chronic ankle instability
vTTS: Vertical time to stabilization
DLL: Double leg landing
CoP: Center of pressure
TTS: Time to stabilization
CAIT: Cumberland Ankle Instability Tool
SV: Sway velocity
ML: Mediolateral
AP: Anteroposterior
vGRF: Vertical ground reaction force
